# Femoroacetabular impingement is more common in military veterans with end-stage hip osteoarthritis than civilian patients: a retrospective case control study

**DOI:** 10.1186/s40779-019-0218-5

**Published:** 2019-08-23

**Authors:** Kate N. Jochimsen, Cale A. Jacobs, Stephen T. Duncan

**Affiliations:** 1 Department of Rehabilitation Sciences, University of Kentucky, 900 South Limestone Street, Lexington, KY 40536 USA; 2 Department of Orthopaedic Surgery and Sports Medicine, University of Kentucky, 740 South Limestone Street, Lexington, KY 40536 USA

**Keywords:** Femoroacetabular impingement, Osteoarthritis, Hip, Acetabular labral tear, Hip arthroplasty

## Abstract

**Background:**

The purpose of this study was to compare the frequency of femoroacetabular impingement (FAI) between matched groups of military veterans and civilian patients with end-stage hip osteoarthritis (OA).

**Methods:**

Patients who underwent a primary total hip arthroplasty (THA) between January 1, 2015 and December 31, 2015 at a single Veteran’s Affairs Hospital were identified. Veterans were then matched 1:2 with civilian patients from our prospective outcome registry. The alpha angle and lateral center-edge angle (LCEA) were measured by a single evaluator. Independent *t*-tests were used to compare joint angles, and Fisher exact tests were used to compare the prevalence of cam (alpha angle ≥60°), pincer (LCEA ≥40°), or mixed-type pathologies.

**Results:**

Twenty-one veterans were matched 1:2 with civilian patients. The mean alpha angle did not significantly differ between groups (*P* = 0.33) nor did the prevalence of cam deformities (*P* = 0.79). The LCEAs were significantly greater in veterans than in civilians (*P* = 0.04), and veterans also demonstrated a significantly greater prevalence of pincer and mixed-type deformities than civilians (*P* = 0.025 and *P* = 0.004, respectively).

**Conclusion:**

These results suggest that FAI is perhaps a more common mechanism in the progression of OA in a veteran population than in a civilian population, as pincer and mixed-type deformities were significantly more common among veterans than civilians. The forces borne by the hip during military training exceed normal physiologic conditions. In addition, the time between symptom onset and surgical correction may be 10–12 months longer for active military personnel than for civilians. The combination of increased physical demands and a protracted time to treatment highlights the need for better recognition of FAI in military members. Future studies are necessary to determine whether earlier intervention may prevent or delay the progression to end-stage OA and the need for total hip arthroplasty.

## Background

Femoroacetabular impingement (FAI) is a hip condition characterized by abnormal bone growth on the articulating surfaces of the femoral neck and acetabulum. During functional activities, this extra bone growth results in supraphysiologic stresses to the anterosuperior acetabular labrum, often leading to labral tears [1]. In active people, tears to the acetabular labrum often result in hip pain and, as some research suggests, accelerated osteoarthritis (OA) [2, 3]. Hip joint conditions such as FAI are more common in highly active populations [4–7]. The stresses placed on the hip joint during high-level sports are similar to those experienced by military personnel during training and active duty [8, 9].

Due to the high physical demands of military training, active military members are at risk for the pathomechanic process of FAI, potentially even at a rate disproportionate to that of civilians. Military personnel over the age of 40 have been reported to be twice as likely to develop OA than the general population [10]. The prevalence of OA in military members has been reported as 28% [11] in contrast to 12% [12] in civilians. As the prevalence of post-traumatic osteoarthritis (PTOA) has increased over the last 10 years, it now represents the most common cause of military disability [11]. In fact, disability is the end result for 40% of military members with FAI who undergo hip arthroscopy [13]. Establishing a causal relationship between FAI and PTOA may enable intervention at an early stage in young, active military members with FAI and symptomatic labral tears.

While FAI has been identified as a causative factor in the development of hip OA in active populations, there have not been any studies to date that have compared the prevalence of FAI in military veterans and in their civilian counterparts. To better elucidate the potential connection among activity level, physical demand, and FAI in the progression of hip OA, the purpose of this retrospective study was to compare the frequency of FAI deformity between matched groups of military veterans and civilian patients with end-stage hip OA. We hypothesized that FAI would be significantly more common in military veterans than in civilians.

## Methods

We performed an Institutional Review Board (IRB)-approved, retrospective review of all patients undergoing a primary total hip arthroplasty (THA) for symptomatic hip osteoarthritis at a single Veteran’s Affairs Hospital between January 1, 2015 and December 31, 2015. After excluding those undergoing a THA secondary to dysplasia, avascular necrosis, fracture, or inflammatory arthritis, we used our IRB-approved, prospective total joint arthroplasty outcomes registry to identify a group of civilian patients who underwent a THA at an academic hospital. Civilian and military veteran patients were matched 1:2, based on sex, age (±5 y), and body mass index (BMI) (±5 kg/m^2^).

Standardized preoperative evaluations included anteroposterior (AP) pelvis and frog-leg lateral radiographs [14, 15]. Morphologic features that were examined on the AP radiograph included the lateral center-edge angle (LCEA) of Wiberg [16]. The parameters used for evaluation of possible femoral head-neck junction abnormalities included the alpha angle (frog-leg lateral) [15, 17, 18]. These measurements were performed only if an appropriate pelvic tilt was present (i.e., sacrococcygeal distance for males 15–50 mm, females 30–65 mm) [19]. These measurements were made using McKesson computer-assisted radiographic measurement software (Emageon, Birmingham, AL, USA). Radiographic analysis was performed by one author (KNJ) with a PhD and experience in radiographic measurements of prearthritic hips. This author (KNJ) received training in radiographic measurements from a board-certified orthopedic surgeon with fellowship training in hip preservation and reconstruction (STD). The intraclass correlation coefficients (ICCs) for intraobserver variability were established prior to the start of this study. The author measured alpha angles and LCEAs on a set of 15 X-rays unrelated to this study at two separate time points. Using those measurements, the ICCs were calculated and ranged from 0.89 to 0.91.

### Statistical analysis

Following the Shapiro-Wilks test for normality, independent *t*-tests were used to compare alpha angles and LCEAs between groups. Fisher’s exact tests were used to determine if the prevalence of cam deformities (alpha angle ≥60°) [20], pincer deformities (LCEA ≥40°) [17], or a mixed deformities (alpha angle ≥60° and LCEA ≥40°) differed between groups. Significance was set a priori at *P* < 0.05 for all tests. An a priori power analysis was not conducted; instead, data were requested for one calendar year.

## Results

Twenty-four military veterans were identified; however, three military veterans were unable to be successfully matched. Twenty-one military veterans were successfully matched 1:2 with civilian patients, resulting in a final sample of 63 patients (21 military veterans and 42 matched civilian patients). The matching process was successful, as the two groups did not differ in age, sex, or BMI (Table 1).

**Table 1:** Demographics and radiographic measures for civilians and military veterans. Continuous variables were compared between civilians and military veterans using independent *t*-tests, and categorical variables (i.e., sex) were compared using Fisher’s exact tests

Item	Civilians (*n* = 42)	Military veterans (*n* = 21)	*P*-value
Female/Male	4/38	2/19	0.99
Age (year)	61.0 ± 9.1	61.0 ± 11.0	0.74
BMI (kg/m^2^)	30.7 ± 5.4	29.9 ± 4.8	0.44
Alpha angle (°)	61.1 ± 11.5	64.3 ± 13.2	0.33
LCEA (°)	33.8 ± 6.8	37.9 ± 8.3	0.04^*^

*BMI* Body mass index, *LCEA* Lateral center-edge angle of Wiberg. ^*^Indicates statistically significant at the 0.05 level

**Fig. 1:**
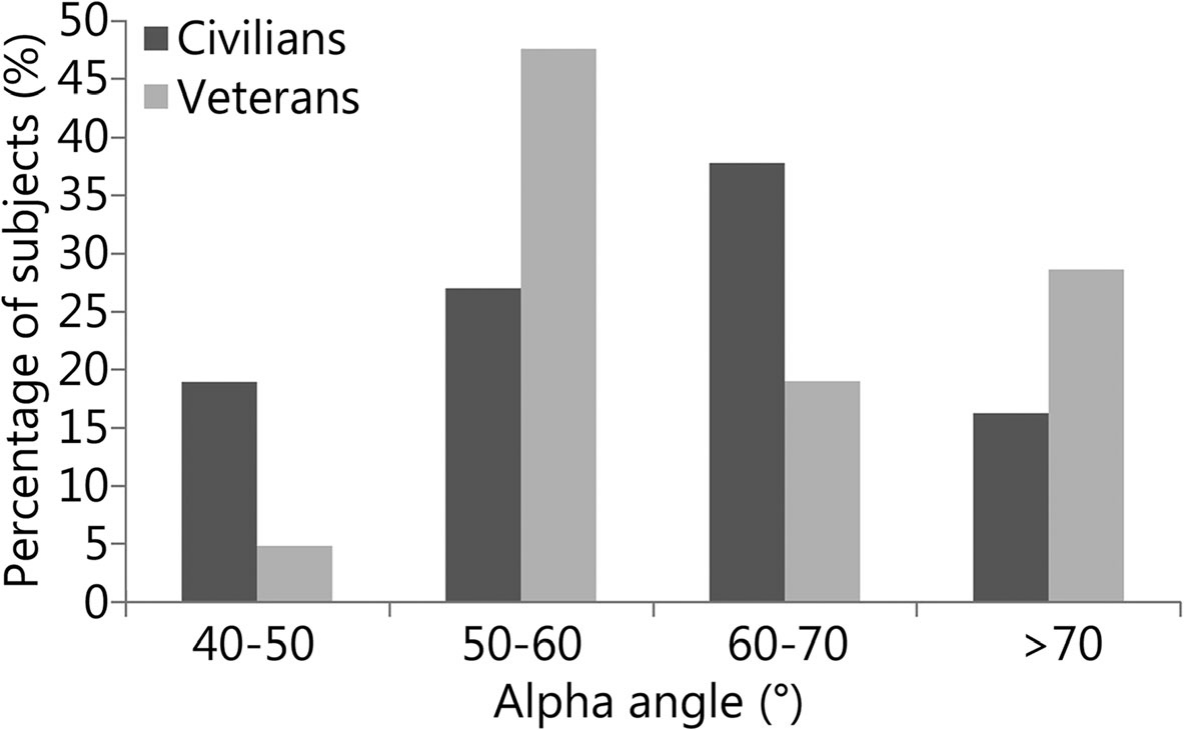
The figure below describes the alpha angle distributions for the civilian and military veteran groups. Alpha angles were measured from preoperative frog-leg lateral radiographs

**Fig. 2:**
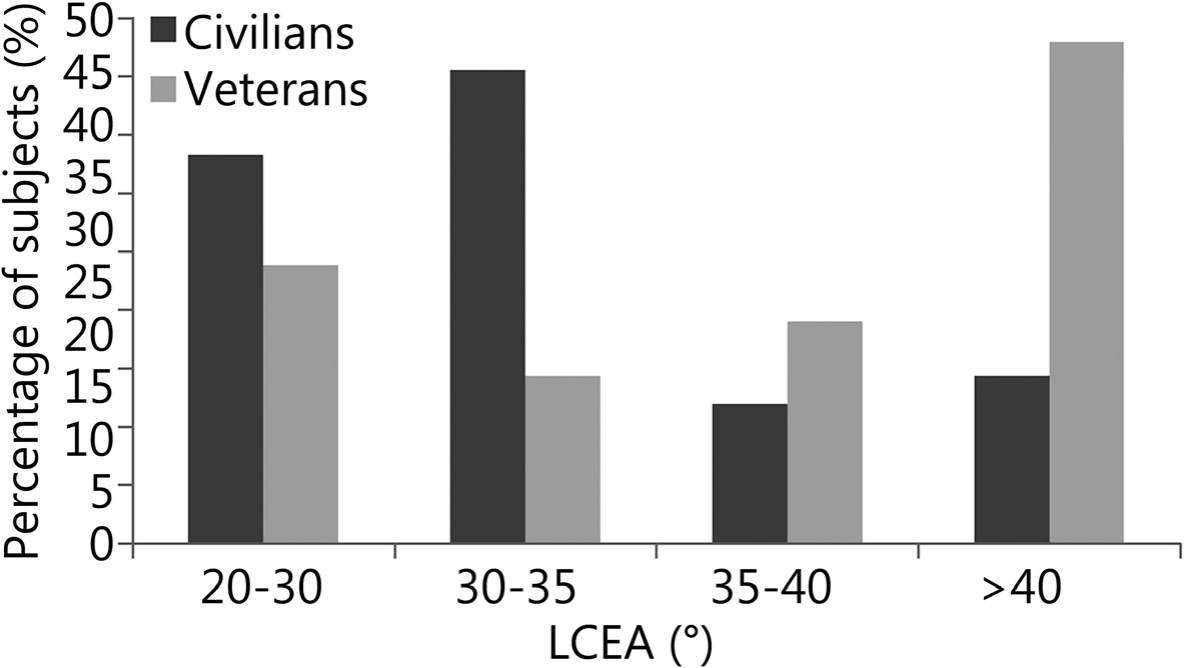
The figure below describes the lateral center-edge angle (LCEA) of Wiberg distributions for civilian and military veteran groups. LCEAs were measured from preoperative anteroposterior pelvis radiographs

**Fig. 3:**
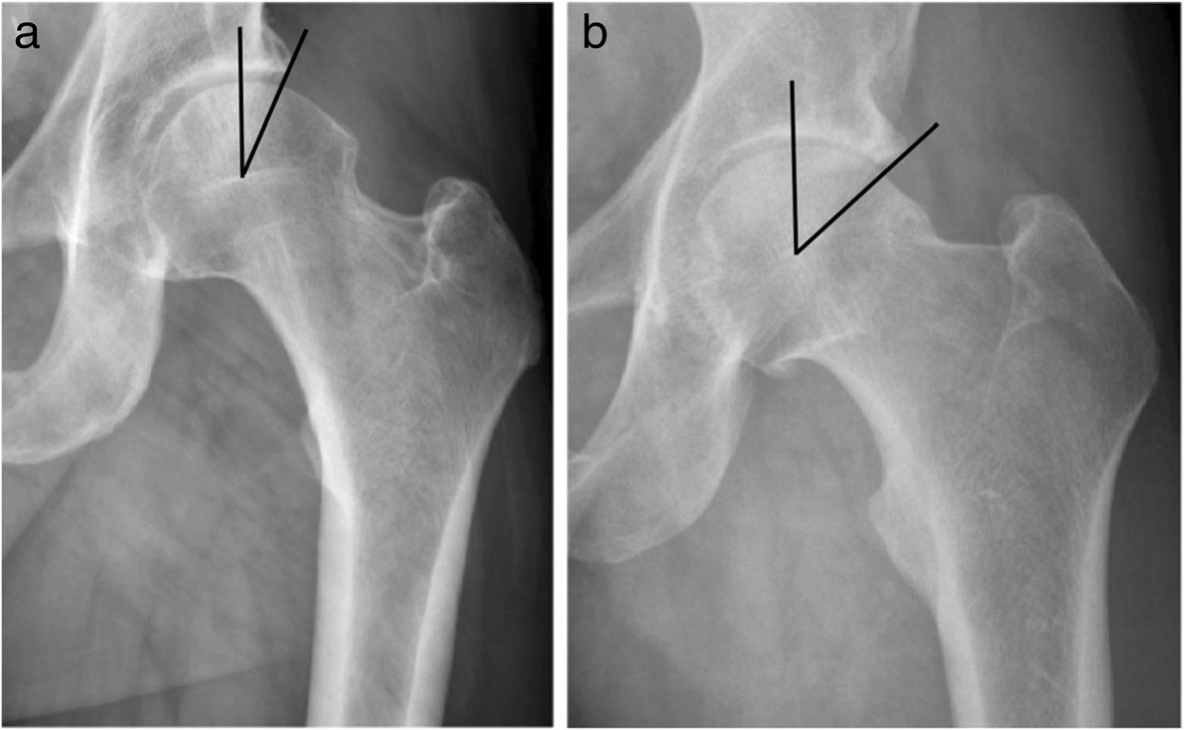
The figure below contains a side-by-side comparison of preoperative anteroposterior pelvis radiographs used in this analysis. The radiograph on the left **a** demonstrates a normal lateral center-edge angle (LCEA) of Wiberg (< 40°), and the radiograph on the right **b** demonstrates a pincer deformity (LCEA ≥40°). Military veterans had significantly greater LCEAs compared with those of civilians using an independent t-test (*P* = 0.04). Military veterans also demonstrated a significantly greater prevalence of pincer deformities when compared with civilians using Fisher’s exact tests (*P* = 0.025)

Distribution data for alpha angles and LCEAs can be visualized in Figs. 1 and 2. Cam-type impingement did not differ between groups (mean alpha angle: military veterans = (64.3 ± 13.2) °, civilians = (61.1 ± 11.5) °, *P* = 0.33, Table 1). When patients were stratified based on the presence of a cam-type deformity, 47.6% (10/21) of military veterans had cam deformities compared to 54% (20/37) of civilians (*P* = 0.79, Table 2). LCEA angles were significantly greater in military veterans (military veterans = (37.9 ± 8.3) °, civilians = (33.8 ± 6.8) °, *P* = 0.04, Table 1), and military veterans also demonstrated a significantly greater prevalence of pincer deformities (military veterans = 9/21, 42.9%, civilians = 6/42, 14.3%, *P* = 0.025, Table 2) and mixed-type deformities (military veterans = 6/21, 28.6%, civilians = 1/42, 2.4%, *P* = 0.004, Table 2) than civilians. Figure 3 contains side-by-side anteroposterior radiographs used in this analysis: one of a normal LCEA (< 40°) and the other of a pincer deformity (LCEA ≥40°).

**Table 2:** Prevalence of structural deformities in the civilian group, military veteran group, and all patients combined, reported as [*n* (%)], and the *P*-value for Fisher’s exact tests comparing the proportion of patients in the civilian and military veteran groups

Structural deformity	Radiographic criteria (°)	Percentage (%)			*P*-value
		Civilians	Veterans	Total	
Cam FAI	AA ≥60	54.0 (20/37)	47.6 (10/21)	51.7 (30/58)	0.786
Pincer FAI	LCEA ≥40	14.3 (6/42)	42.9 (9/21)	23.8 (15/63)	0.025^*^
Mixed FAI	AA ≥60 and LCEA ≥40	2.4 (1/42)	28.6 (6/21)	11.1 (7/63)	0.004^*^

*FAI* Femoroacetabular impingement, *AA* Alpha angle, *LCEA* Lateral center-edge angle of Wiberg; ^*^Indicates statistically significant at the 0.05 level

A post hoc power analysis for the primary hypothesis that FAI would be significantly more common in military veterans than in civilians was conducted. Using Fisher’s exact tests, we had sufficient power to find significant differences in the prevalence of mixed-type deformities between military veterans and civilians (86.5% power and an alpha level of 0.05).

## Discussion

As a group, active military members are likely prone to FAI secondary to the high physical demands of their jobs. Previous studies have demonstrated improvement in pain and function following arthroscopic treatment of FAI among military members; however, there are mixed results on whether these individuals return to active duty [13, 21]. The reasons for this are not fully understood, and thus, further evidence to properly diagnose and direct treatment for this population is needed.

One potential cause for continued pain is the prevalence of hip OA. We therefore sought to determine if there was a clinically significant difference in the frequency of FAI deformities between matched groups of military veterans and civilian patients with end-stage hip OA. Contrary to our hypothesis, we found that there was no difference between the two cohorts regarding their mean alpha angles or the prevalence of cam deformities; however, mean LCEA and the prevalence of both pincer and mixed-type deformities were significantly greater in military veterans than in their matched civilian counterparts. These findings are consistent with the literature describing a higher incidence of symptomatic FAI in athletic populations [4–7]. Furthermore, pincer impingement, unlike cam deformities, results in circumferential cartilage damage and labral ossification [22]. Though causality cannot be implied from this cross-sectional study, an association between activity level and extent of bony deformity has been repeatedly demonstrated [23–25].

As posttraumatic OA now represents the most common cause of military disability [11], the findings of this study support that there may be value for earlier, more aggressive interventions for military members with symptomatic FAI to help prevent or halt the progression of OA. While it may be plausible that few, if any, of these veterans had symptomatic FAI during their years of active duty, recent evidence suggests that symptomatic military personnel have to wait upwards of 28 months prior to appropriate intervention [26]. Additional evidence suggests that FAI deformities may develop early on in life [23]. Due to the physical demands of military jobs, return to active duty following hip arthroscopy can be difficult and is multifactorial [13]. It has yet to be seen if decreasing the patient’s duration of symptoms with earlier intervention can improve the percentage of those returning to active duty and/or alter the progression to posttraumatic OA.

### Limitations

Our study has some limitations. First, radiographic measures of FAI are potentially unreliable between and within individual readers. To address this concern, one author with good intrarater reliability (ICC 0.89–0.91) made all measurements, thereby eliminating the potential confounder of poor interrater reliability; however, the external validity of these findings may be impeded secondary to the inherent limitations with these radiographic measures. Another limitation of this study was the small sample size. No a priori power analysis was conducted because we aimed to include all military veterans that underwent a THA at a single Veteran’s Affairs hospital in a single calendar year. It was unexpected that only 21 military veterans would meet our inclusion criteria. Unfortunately, requesting data from additional years was not feasible, and therefore, this analysis was limited. A post hoc power analysis revealed that we had sufficient power to analyze the primary aim; however, due to the small sample size, these results should be interpreted cautiously. Due to the retrospective nature of this study, activity level, job description, military branch, and years in service were unavailable for analysis. Though we cannot say with certainty that those patients in the veterans affairs (VA) cohort were exposed to increased physical demands, the inherent nature of military training would lend itself to this idea. Finally, this analysis of patients with end-stage hip OA only included preoperative data, and as such, causality cannot be established. Future longitudinal studies of active duty military personnel are necessary to confirm the role of pincer-type deformities on the progression of posttraumatic OA in this active patient population.

With current literature suggesting that disability is the end result for approximately 40% of military personnel with FAI undergoing hip arthroscopy, further research is needed to determine what factors are influencing this outcome [13]. Further research is necessary to examine the causality behind symptomatic FAI and the impact of an extended duration of symptoms on joint health, return to active duty, and patient satisfaction. This study demonstrates that military veterans may be presenting with an injury based more on the posttraumatic OA model seen following hip impingement and subsequent acetabular labral tears than on the traditional path of idiopathic OA. Additional research is necessary to clarify the relationship between activity level and FAI progression and to determine the most appropriate intervention to improve return to active duty in military personnel with FAI.

## Conclusions

The results of this study demonstrate that FAI appears to be a common mechanism in the progression of OA in a military veteran population, as both measures of pincer deformity and prevalence of pincer lesions were significantly greater in military veterans than in their matched civilian counterparts. The forces borne by the hip during military training often exceed normal physiologic conditions due to the extreme joint ranges of motion and the frequency of intense, dynamic activity. In addition, it has been reported that the time between the onset of symptoms and surgical correction may be 10–12 months longer for active duty military personnel than for civilians [26, 27]. The combination of increased physical demands and a protracted time to diagnosis and treatment highlights the need for better education and recognition of FAI in military members. Future studies are necessary to determine whether earlier diagnosis and surgical correction of FAI may prevent or delay the progression of OA in physically active patients.

## Data Availability

The datasets used during the current study are available from the corresponding author upon reasonable request.

## Abbreviations

AP: Anteroposterior
BMI: Body mass index
FAI: Femoroacetabular impingement
ICCs: Intraclass correlation coefficients
LCEA: Lateral center-edge angle
OA: Osteoarthritis
PTOA: Post-traumatic osteoarthritis
THA: Total hip arthroplasty
VA: Veterans affairs
